# The Temporal and Spatial Changes of Health Inequality in Rural China

**DOI:** 10.3389/fpubh.2022.821384

**Published:** 2022-02-10

**Authors:** Jinqi Jiang, Wanzhen Huang, Yunru Liu, Zhenhua Wang

**Affiliations:** ^1^College of Economics and Management, Shenyang Agricultural University, Shenyang, China; ^2^College of Economics and Management, China Agricultural University, Beijing, China; ^3^College of Innovation and Entrepreneurship, Quzhou University, Quzhou, China

**Keywords:** health inequality, Rural China, health utility index, interval regression, concentration index

## Abstract

This article estimates the temporal and spatial changes of health inequality in rural China from 2010 to 2018. Based on a panel database of 29,616 rural residents, the Health Utility Index (HUI) and a spatial econometric model are used for analysis. The results show that, on the temporal dimension, the health inequality of rural China first expands and then deflates. On the spatial dimension, the health inequality gradually deflates from eastern to western China. Furthermore, from 2010 to 2018, the high and low-value areas constantly changed among different provinces. After decomposing the causes of health inequality, it is found that behind the health inequality is the difference of socioeconomic-related status. Moreover, narrowing the difference in socioeconomic-related status is the key to improving health inequality.

## Introduction

Since China's so-called “reform and opening up” policy was implemented, China's economy has grown rapidly. China's real gross domestic product (GDP) in 2021 was more than 219 times of its real GDP in 1980 (International Monetary Fund, 2021). At the same time, people's health in China also has significant improvements. According to the WHO's Global Health Estimates, the life expectancy in China has increased from 33 to 77 years old during the last 35 years. However, observers have noted that a wealthier China has increased health disparities (1). Indeed, due to the living conditions and the limiting health resources, the health inequality does not occur only between the urban and rural, but also between the people inner the rural which is more serious (2–4). The WHO proposes that the scope of health covers not only medical and health but also the fairness related to social-economic development (5). Health inequality not only prevents the right to health of people (6), but also the realization of the sustainable development. In order to achieve health equality, it is vital to explore the recent trend of health inequality and determine the causes.

According to the existing literature, since the 1990s, the health inequality has existed in the whole of China (4, 7). It is found that there exists pro-rich in health and utilization of healthcare (2, 8), income contribution to 13–20% inequality of healthcare utilization (9, 10). That means the health inequality between the poor and the rich is not a reflection of their different preferences, but the constraint conditions, lower income, less access to health insurance, and living conditions that are more likely to encourage the spread of disease (11–13). Moreover, both rural and urban health inequalities are increasing in China, as in rural areas is larger than that of in urban areas (4, 12, 14). As an important component of human capital, health is determined not only by micro factors but also by spatial factors (15). Behind the spatial factors, there are differences in the dietary habits, economy, health security, etc., which means exploring the causes of the health inequality the inter-regional interactions could not be neglected. However, most literature explored the health inequality in China from the temporal perspective and using the database either from the early period (i.e., 1990–2006) or from a specific area, it is necessary to consider the new changes during the decades. Besides, the spatial evidence is less proven.

Furthermore, from the methodology perspective, most literature employs self-assessment health (SAH) status to measure the health inequality. However, due to the SAH is the ordered responses, which are ordinal data expressing rankings rather than cardinal numbers, the index to measure the health inequality (i.e., Gini coefficient and Theil index, etc.) can no longer be meaningfully calculated (14). In this paper, we apply the approach developed in Wang et al. (3), to transition the order SAH to cardinal Health Utility Index (HUI). We also followed Baeten et al. (9) and resort to an interval regression model to identify the factors that are likely to be responsible for the dynamics in rural China's health inequality over the period. In order to explore the temporal and spatial changes of health inequality in rural China, this paper uses a panel database of 29,616 rural residents from 2010 to 2018 to analyze the temporal and spatial changes of health inequality in rural China. On the basis of constructing the HUI to reduce the measurement bias and using the Centralized Index (CI) to measure health inequality of the Chinese rural residents, we use Moran's global index to explore the spatial variations.

The remainder of this paper is organized as follows. In the next section, we briefly introduce the measurement methods of health inequality. Next, the data sources and variables are described in detail. We then explore the temporal and spatial changes of health inequality in rural China. Finally, we turn to the results and put forward the policy recommendations.

## Materials and Methods

### Measurement of Health Inequality

One of the challenges in investigating health inequality is that health information is only available at an ordinal level. For instance, the health information from the database used in this study is the simple question, “how is your health in general?,” with response categories ranging from “very good” or “excellent” to “poor” or “very poor.” However, the ordinal health status does not provide a cardinal health (utility) scale that can be used, it creates a problem for the measurement of health inequality (16). The health concentration index, and the related slope index of inequality, requires information on health in the form of either a continuous variable or a dichotomous variable (17). However, the dichotomous variables impose some sort of scaling assumption arbitrarily and have well-known disadvantages (17).

This paper constructs the ordinal HUI to measure the health inequality in China by interval regression, which is able to transform the ordinal variables to continuous when the values of the upper and lower limits of the intervals are known. Differing from the previous studies that used the Canadian threshold directly, we used the Simulated Visual Score (VAS) cumulative distribution intervals, which are derived from the Chinese. It can provide more accurate evidence for China. As for the measurement of health inequality, this paper concerned the concentration index, which is widely used for measuring inequality, especially socioeconomic-related health inequality (1).

In conclusion, the most important advantages of the methodology in this paper are as follows, we constructed a more realistic cardinal variable HUI to measure the health inequality in China. Based on the health status intervals of Chinese rather than Canadian threshold directly (which is widely used in existing literature), this paper can measure the Chinese health inequality more accurately. However, this paper assumed the health of all Chinese no matter from the urban or rural is in conformity to the same distribution. That may be a strong assumption, and if the model puts forward to “different conditions,” it would be more realistic.

#### From the SAH to the HUI

As mentioned above, this paper constructed the ordinal HUI to measure the health inequality, which is resorted to the interval regression model to transform ordinal SAH values to cardinal values. The details of the processes are as follows:

First of all, before the interval regression, we matched the SAH with the health thresholds, which are appropriate for China. Differing from the previous studies that used the Canadian threshold directly (16), we used the VAS cumulative distribution threshold, which was proposed by Baeten et al. derived from the Chinese (9). According to reality of the database and the VAS threshold, the SAH value was turned into the following four types of interval threshold, these are “Excellent health” = (0.91, 1), “Very health” = (0.80, 0.91), “Health” = (0.50, 0.80), and “Unhealthy” = (0, 0.50). After being matched with the threshold, the HUI can be fitted by the regression estimation result of equation (1):


(1) $$\begin{array}{l} {\text{HUI}}_{\text{it}}={\text{a}}_{\text{it}}+\sum \beta_{\text{it}}{\text{X}}_{\text{it}}+\sum \gamma_{\text{itit}}+\mu_{\text{it}} \end{array}$$

where, *HUI*_*it*_ is the intervals of SAH, *a*_*it*_ is the constant term, *X*_*it*_ is a series of explanatory factors of health that include income, education, family assets, etc. **it** contains the individual variables, such as age, gender, marital status, family size, political participation, and region. Moreover, *μ*_**it**_ is an error term.

#### Measurement of Health Inequality

The health inequality in this paper refers to the social-economic status, that is, the systematic external differences due to different socioeconomic status (13). The health inequality in this paper is measured by three specific indicators, these are the assets of the individual's family, the individual's income, and education level.

In this paper, we used the CI to measure the health inequality and based on the decomposition of the CI to illustrate the source of health inequalities. The CI can not only reflect the health inequality related to the social-economic status, but also flexibly reflect the distribution changes. There are many calculation methods for the CI. The formula to calculate the CI is shown in equation (2):


(2) $$\begin{array}{l} \text{C}=\frac{2}{\text{n}\mu }\sum_{\text{i}=1}^{\text{n}}{\text{y}}_{\text{i}}{\text{R}}_{\text{i}}-1 \end{array}$$

In this formula, n is the number of observations, **μ** is the mean of the HUI, and *y*_*i*_ is the factors of the socioeconomic status. $R_{i}=\frac{i}{n}$ is the order of individuals in income, family assets, or education levels.

Then convert formula (2) into the following form:


(3) $$\begin{array}{l} \text{C}=\frac{2}{\mu }co\text{v}\;({\text{y}}_{\text{i}},{\text{R}}_{\text{i}}) \end{array}$$

Here, *cov* is the covariance of *y*_*i*_ *and R*_*i*_.

The CI value is usually between −1 and 1. If the CI is positive, that means there is a “pro-rich” healthy inequality, which is to say, healthy capital is more concentrated in individuals with higher socioeconomic status. If the CI is negative, the health inequalities are “pro-poor.” Individuals with low socioeconomic status have more health capital. When the CI equals to 1 or −1, it is an extreme state indicating that healthy capital is completely held by individuals with high socioeconomic status or individuals with low socioeconomic status. When the concentration index is 0, it means that healthy capital has achieved an equal distribution between the socioeconomic status of high and the low.

### Spatial Autocorrelation Test

We used the global Moran's I to test the spatial autocorrelation of health inequalities that vary in provinces. The formula to calculate the Moran's I is shown in equation (4):


(4) $$\begin{array}{l} \text{I}=\frac{\text{n}}{{\text{S}}_{0}}\frac{\sum_{\text{i}=1}^{\text{n}}\sum_{\text{j}=1}^{\text{n}}{\text{w}}_{\text{i},\text{j}}{\text{z}}_{\text{i}}{\text{z}}_{\text{j}}}{\sum_{\text{i}=1}^{\text{n}}{\text{z}}_{\text{i}}^{2}} \end{array}$$

Where *z*_*i*_ is the deviation between the attribute of i and its mean, *w*_*i, j*_ is the spatial weights between i and j, n is the number of the factors, *S*_0_ it's the aggregation of all the spatial weights.

## Data Sources and Variable Descriptions

### Data Source

The database of this paper is from the China Family Panel Studies (CFPS). The CFPS is a nationwide large sample micro-survey database in China, which covered social, economic, demographic, health, and education aspects. The CFPS survey began a sampling survey at both the individual and the household levels based on random sampling since 2010 and comes out every 2 years, the latest round of surveys completed in 2018. We selected rural sample data from 2010 to 2018. Before the analysis, we excluded the samples with missing information and numerical anomalies on important variables, such as personal income, health status, demographic characteristics, family economic characteristics, and provinces. After data cleaning, the entire sample is of 29,616 rural individuals.

### Variable Definition and Description

In order to estimate the temporal and spatial changes of health inequalities more precisely, this paper mainly measures individual income indicators, such as income, education, and family assets. According to the database, the income used in this paper is mainly composed of the individual's annual income. Moreover, here we take the logarithm form.

In order to estimate the spatial feature of health inequality, this paper used the information of 25 provinces in mainland China. Provincial dummy variables can further control unobservable regional effects, such as production habits. Furthermore, this paper estimated the region-level health inequality by dividing the eastern, central, and western regions of mainland China. The specific variable settings and descriptions are shown in Table 1.

**Table 1:** Variable definition and statistical description.

**Variables**	**Definitions**	**Mean**	**S.D**.	**Max**	**Min**
Income	Logarithm of the total annual income of the individual	9.44	1.5893	0	16.15
Family assets	Logarithm of personal family assets	11.46	2.1818	0	17.34
Education	Individual years of schooling	8.45	4.6070	0	22
Age	Respondent age	40.57	13.0803	14	96
Family size	Numbers of family population	4.28	1.8594	1	26
Gender	1 = Male, 0 = Female	0.57	0.4954	0	1
Marital status	1 = Married, 0 = Other	0.80	0.3989	0	1
Political participation	1 = Communist Party of China (CPC) member; 0 = Other	0.07	0.2619	0	1
Province	25 provinces in mainland China	-	-	-	-
Region	1 = Western China, 2 = Center China, 3 = Eastern China	2.23	0.8096	1	3
Self-assessment health	1 = Unhealthy, 2 = General health, 3 = Health, 4 = Very health, 5 = Excellent health	2.48	1.2005	1	5

**Figure 1:**
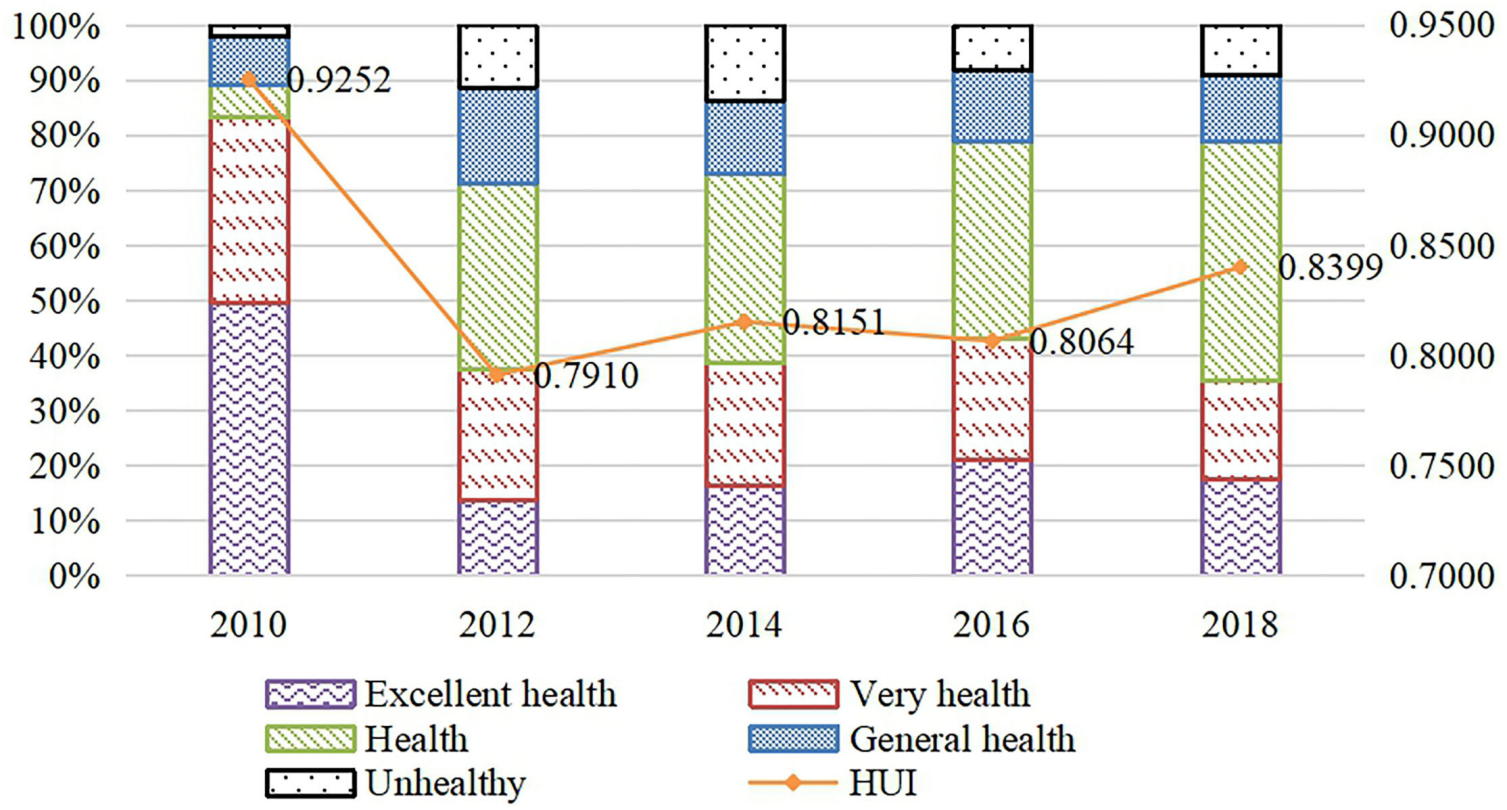
SAH and HUI from 2010 to 2018. HUI, Heath Utility Index; SAH, self-assessment health.

Before the empirical analysis, this section describes the health status in rural China. As shown in Figure 1, from 2010 to 2018, Chinese rural residents' health status first deteriorates and then improves. From 2010 to 2018, the HUI values are 0.9252, 0.7910, 0.8151, 0.8064, and 0.8399. In addition, the proportion of “Unhealthy” is shown a fluctuant downward trend. It can be seen that the overall health status of the sample group has fluctuated during the 8 years.

As shown in Table 2, from a regional perspective, from 2010 to 2018, the HUI in eastern, central, and western China shows a rising trend. First, to the horizontal comparison, from 2010 to 2018, the health status in the western region is lower than that in central or eastern China. That indicates that the health of rural residents in China got worse from east to west. Overall, the mean values of the sample health indicators in the central region and the eastern region are relatively close, but there are also differences. From 2010 to 2014, the health status in the central China is better than that in eastern China. From 2014 to 2018, the health status in the eastern region was improved rapidly. In 2018, the health of the sample in the eastern region is the best among the three regions.

**Table 2:** HUI of the eastern, middle, and western China from 2010 to 2018.

	**2010**	**2012**	**2014**	**2016**	**2018**
Western	0.9210	0.7799	0.7981	0.7971	0.8331
Central	0.9267	0.8021	0.8255	0.8067	0.8382
Eastern	0.9259	0.7935	0.8206	0.8109	0.8450

*HUI, Heath Utility Index*.

## Temporal and Spatial Changes of Health Inequality in Rural China

### The Overall Trend of Health Inequality: From the Temporal Perspectives

Based on the interval regression, we transformed the SAH to HUI, the results are shown in Table 3. Table 3 shows that income has a significant positive effect on HUI. When time fixed effect and province fixed effect are controlled, income increases 1 unit and HUI increases 0.008. There is also a positive effect of family assets on HUI, when family assets increase one unit and HUI increases 0.002. Age has a negative effect on HUI. Health, as a human capital, continues to decrease with the increase of age, and the investment demand of elderly rural residents on health will also decline. In rural China, the health status of men is better than the women. Education plays a positive role in promoting the health of rural residents, and the improvement of health status is monotonically increasing with the education level. Stable marriage status has a positive effect on the rural residents' health, which indicates that stable marriage status is conducive to maintaining the health of rural residents. Furthermore, the rural residents who participated in Communist Party of China (CPC) are healthier than others. In addition, there are more populations in the family, the healthier the individual is.

**Table 3:** The estimation of interval regression.

	**Coef**.	**S.E**.
Income	0.008^***^	(0.001)
Family assets	0.002^***^	(0.000)
Age	−0.006^***^	(0.002)
Age2	0.000	(0.000)
Gender-male	0.022^***^	(0.002)
Education	0.002^**^	(0.000)
Marital status-married	0.006^**^	(0.003)
Political participation-CPC	0.012^***^	(0.004)
Region-central	−0.019	(0.091)
Region-eastern	−0.012	(0.093)
Familysize	0.001^***^	(0.000)
Time fixed	YES	
Province fixed	YES	
_cons	0.965^***^	(0.091)
sigma_u		
_cons	0.069^***^	(0.001)
sigma_e		
_cons	0.110^***^	(0.001)

*SEs in parentheses*,

** *p < 0.01*,

*** *p < 0.001*.

Further, in this section, we calculate the CI to measure the health inequality related to socioeconomic status. The result is shown in Table 4. According to Table 4, from 2010 to 2018, the CIs of HUI are 0.0155, 0.0436, 0.0552, 0.0381, and 0.0234, which indicates that the health inequality does exist in rural China. Moreover, it expands from 2010 to 2012 and then deflates from 2014 to 2018, which means the health inequality has been improving since 2014.

**Table 4:** CI of health inequality in rural China from 2010 to 2018.

**Index**	**2010**	**2012**	**2014**	**2016**	**2018**
CI—HUI	0.0155	0.0436	0.0552	0.0381	0.0234
CI—income	0.0081	0.0251	0.0396	0.0160	0.0076
CI—education	0.0085	0.0276	0.0361	0.0195	0.0115
CI—household assets	0.0031	0.0069	0.0078	0.0058	−0.0010

*CI, Centralized Index*.

In order to have a more distinct view of the changes of health inequality of rural residents in China, this section also provides a time trend of the CI of economic-related status variables between 2010 and 2018. As it is shown in Table 4, there are wide disparities in the health status of different social groups. First, from 2010 to 2018, the CIs of income are 0.0081, 0.0251, 0.0396, 0.0160, and 0.0076. It indicates that healthy capital is more concentrated in the richer individuals. However, the trend of “pro-rich” inequality gradually reduces. The CI of education is positive from 2010 to 2018. In addition, the values of the CI of the education are larger than others, which indicate that health capital is more concentrated in the highly educated rural residents, and this kind of health inequality is also gradually narrowing. The CI of household assets shows an increasing trend from 2010 to 2014 and a downward trend after 2014, and the value turns negative in 2018. That is to say, in 2018 the health resources are more concentrated in individuals with fewer household assets. One of the probable reasons may be the promoting of China's poverty alleviation strategy and the new rural medical insurance system.

In addition, there are different effects on social-economic variables. Specifically, in 2010–2018, the level of health inequality associated with household assets is much lower than the health inequalities associated with income and education. In 2010–2012, education-related health inequalities are more serious than income-related health inequalities, and the growth rate is faster. Income-related health inequality is more serious than education-related health inequalities, and it expands largely. By 2018, income-related health inequality has declined even more. Moreover, the health inequality which is associated with household assets turned negative in 2018. Finally, the situation of the education-related health inequality is greater than the income-related health inequality with household assets is formed.

### The Overall Trend of Health Inequality: From the Spatial Perspectives

There are regional differences in health status in China (18). In order to explore the spatial variations of health inequality, this section first estimates the health inequalities of rural residents in China among the 25 provinces in China, and then eastern, central, and western China.

According to Table 5, in 2010, the province that suffered the most serious health inequality is Tianjin (津), in which the CI of health inequality is 0.0176. In addition, the high-value areas are concentrated in Sichuan (川), Shanghai (沪), Jiangsu (苏), Hebei (冀), Jilin (吉), Henan (豫), Fujian (闽), Anhui (皖), 219 Guizhou (黔). The low-value areas are dotted in the Heilongjiang (黑), Jiangxi (赣), Guangxi (桂), 220 Zhejiang (浙) and Yunnan (滇). Compared that to 2010, the spatial health inequality has changed, and the overall condition has improved in 2012. The high-value area has been transferred from the coastal areas to the southwestern inland areas, such as Tianjin (津) and Fujian (闽). The low-value area is 223 dotted in the Jiangsu (苏), Heilongjiang (黑) and Chongqing (渝). In 2014, the most serious inequality 224 is happened in Hubei (鄂) rather than Tianjin (津). And the best condition is in Zhejiang (浙), 225 Jiangsu (苏) and Beijing (京). In 2016, the high-value areas of health inequality and the low-value 226 areas are staggered, and the high-value areas are transferred to the Hubei (鄂) in the north China. The 227 low-value areas are mainly distributed in Zhejiang (浙), Jiangsu (苏), and Beijing (京). In 2018, the 228 health inequality get better in every province. And the best three provinces are Shaanxi (陕), 229 Heilongjiang (黑) and Beijing (京). The worst condition occurs in Guizhou (黔).

**Table 5:** CI of health inequality among 25 province in rural China from 2010 to 2018.

	**2010**	**2012**	**2014**	**2016**	**2018**
Beijing (京)	–	–	0.0195	0.0267	0.0109
Tianjin (津)	0.0176	0.0508	0.0476	0.0356	0.0168
Shanghai (沪)	0.0161	0.0445	0.0436	0.0387	0.0192
Jiangsu (苏)	0.0160	0.0375	0.0399	0.0397	0.0148
Guangdong (粤)	0.0141	0.0413	0.0479	0.0342	0.0193
Zhejiang (浙)	0.0132	0.0407	0.0433	0.0369	0.0161
Shandong (鲁)	0.0146	0.0396	0.0551	0.0335	0.0181
Liaoning (辽)	0.0146	0.0403	0.0517	0.0325	0.0187
Fujian (闽)	0.0151	0.0489	0.0502	0.0422	0.0162
Hebei (冀)	0.0154	0.0402	0.0550	0.0301	0.0168
Guangxi (桂)	0.0134	0.0386	0.0457	0.0385	0.0211
Shanxi (晋)	0.0144	0.0394	0.0447	0.0329	0.0169
Jilin (吉)	0.0154	0.0443	0.0475	0.0340	0.0189
Heilongjiang (黑)	0.0139	0.0373	0.0502	0.0304	0.0109
Henan (豫)	0.0153	0.0398	0.0513	0.0321	0.0181
Hubei (鄂)	0.0144	0.0394	0.0638	0.0375	0.0172
Hunan (湘)	0.0140	0.0385	0.0638	0.0372	0.0169
Anhui (皖)	0.0151	0.0407	0.0571	0.0317	0.0195
Jiangxi (赣)	0.0136	0.0401	0.0496	0.0367	0.0183
Chongqing (渝)	0.0142	0.0350	0.0613	0.0243	0.0163
Sichuan (川)	0.0163	0.0417	0.0508	0.0333	0.0206
Guizhou (黔)	0.0149	0.0397	0.0621	0.0334	0.0218
Yunnan (滇)	0.0131	0.0481	0.0505	0.0392	0.0190
Shanxi (陕)	0.0141	0.0402	0.0546	0.0312	0.0128
Gansu (甘)	0.0145	0.0391	0.0538	0.0310	0.0175

*CI, Centralized Index*.

**Figure 2:**
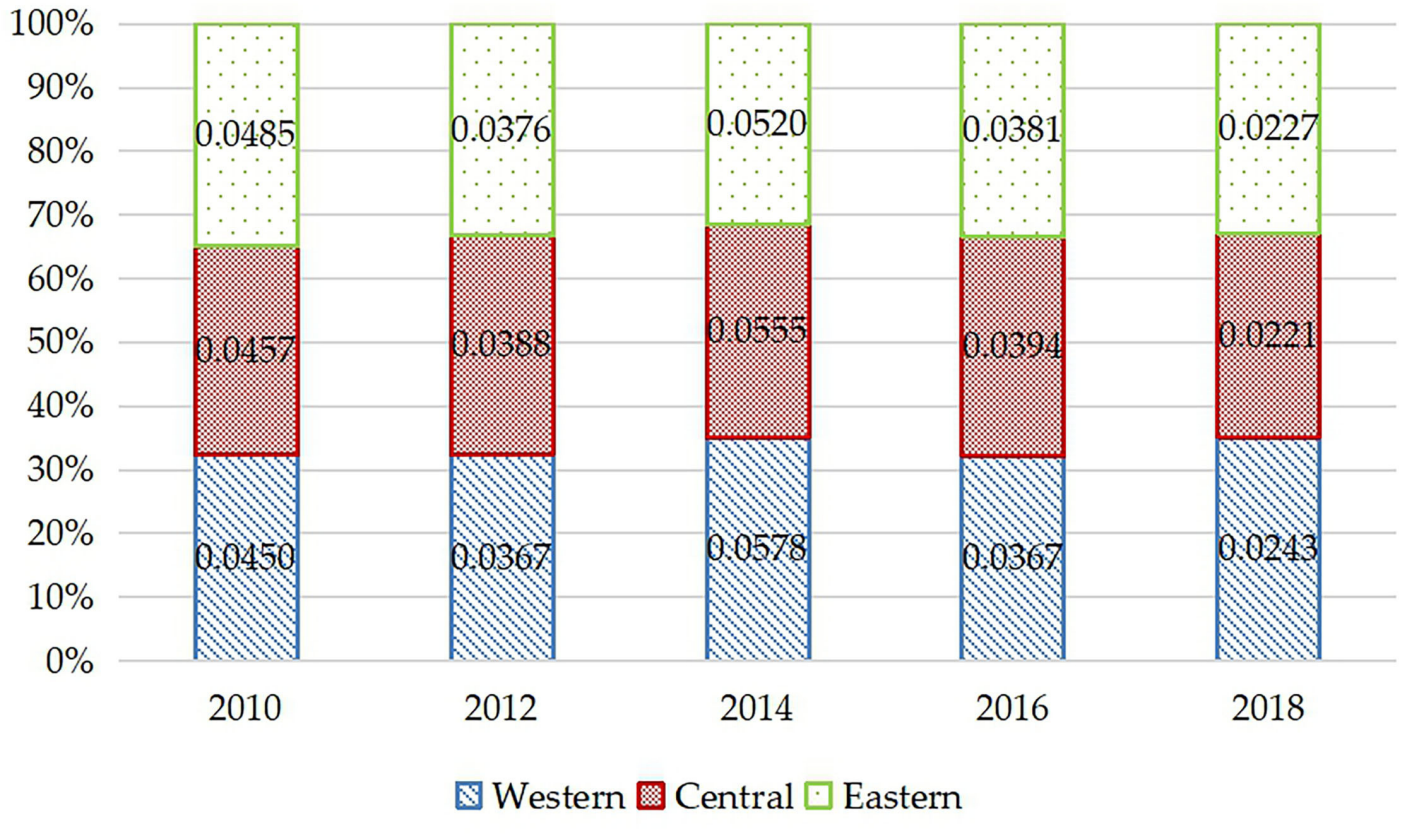
Regional variations of health inequality in rural China from 2010 to 2018.

In this part, we divide the 25 provinces into 3 regions, which is eastern, central, and western China. The regional variations of health inequality are shown in Figure 2. According to Figure 2, from 2010 to 2018, although health inequality widespread in eastern, central, and western China, the overall trend narrows. This process can also be divided into two stages. In 2010–2014, the health inequality in the eastern, central, and western regions shows an expanding trend, but after 2014, the expansion of the eastern region is significantly slowed down. Health inequality in the western region is the most serious. After 2014, inequality in the eastern, central, and western regions shows a trend of continuous decline, and the western region experienced the fastest decline, while the eastern region experienced the slowest decline. By 2018, it shows the trend of diminishing from east, middle to western regions.

### Spatial Correlation Test of Health Inequality

In this section, we conduct a global Moran's Index to test the spatial autocorrelation among different regions. As it is shown in Table 6, the global Moran's Index values of the health inequality of Chinese rural residents during 2010 and 2018 are 0.206, 0.285, 0.268, −0.048, and 0.440. In addition, except the value in 2016, all of the Moran's Index are statistically significant and all of them are greater than 0. The results show that there is a high global spatial positive correlation among the Chinese rural residents during 2010–2014 and 2018. The spatial autocorrelation of health inequality means that there is a kind of agglomeration in spatial conditions. The agglomeration may be caused by two reasons, one is that the provinces close to each other may have a similar natural endowment and similar living conditions. The other one is that economic exchanges between neighboring provinces are more convenient, which promotes the flow of health resources and forms spatial correlation.

**Table 6:** Spatial correlation test of HUI.

	**2010**	**2012**	**2014**	**2016**	**2018**
Moran's I	0.206	0.285	0.268	−0.048	0.440
*P*-value	0.050	0.011	0.008	0.960	0.000

*HUI, Heath Utility Index*.

## Discussion

This study explores the temporal and spatial socioeconomic-related health inequalities among rural China by using the CFPS database from 2010 to 2018. By using the interval regression based on the Chinese threshold to construct the HUI and measure the CI, this paper finds that the CI values of HUI from 2010to 2018 are 0.0155, 0.0436, 0.0552, 0.0381, and 0.0234, which means there is pro-rich health inequality in rural China. In addition, the trend first expands and then deflates. This result is consistent with the finding of existing literature, such as Xie (2), Wang and Yu (10, 14), Xue and Lim (8), who found that there existed significant pro-rich inequality in health in China. Furthermore, we observed that the absolute values of CI in health in this study are lower than those in existing studies using the database from 1990 to 2006. It shows that although health inequality still exists in rural China, it improves from 2010 to 2018.

From the spatial perspective, we find that from 2010 to 2018, the health inequality gradually deflates from eastern to western China. The province of most serious health inequality in China changes from Tianjin (津) to Guizhou (黔), in which the CI value changes from 0.0176 in Tianjin to 0.0218 in Guizhou. It suggests that, although overall the health inequality improves, the difference from provinces should not be neglected. Furthermore, by dividing the provinces into three main regions, we find that although health inequality widespread in eastern, central, and western China, the overall trend narrows. This process can also be divided into two stages. The first stage is from 2010 to 2014, the health inequality in eastern, central, and western regions shows an expanding trend, but after 2014, the expansion of the eastern region significantly slows down. The second stage is in 2014–2018, health inequality in the eastern, central, and western regions steadily decline, and the fastest decline happens in the western region, while the eastern region experienced the slowest decline. By 2018, it shows the trend of diminishing from east, middle to western regions. The results are almost consistent with the finding of Yang et al. (19), but in their paper, they mainly used spatial methods rather than the CI values, the specific values are incommensurable.

Results from the spatial econometric model show that the global Moran's Index values of the health inequality in rural China from 2010 to 2018 are 0.206, 0.285, 0.268, −0.048, and 0.440. In addition, except the value in 2016, all of the Moran's Index are statistically significant and all of them are positive, which means that there exists spatial autocorrelation of health inequality. However, the values in this paper are smaller than the values estimated by Yang et al. (19). The reason might depend on the difference of the objects. The objects in Yang et al. (19) are elderly people in China, as in this paper are rural people.

This paper highlights several recommendations that might be helpful to narrow health inequality in rural China. Firstly, it is quite necessary for the government to realize that there does exist health inequality in rural China. Moreover, behind the health inequality is social-economic inequality. It is important to narrow the gap in household income, especially under the strategy of rural vitalization. Second, the government ought to pay more attention to the health-related quality of life and access to health services among rural people with lower income and less education. Thirdly, it is also necessary to carry out health education. It is helpful to improve healthy awareness to promote healthy lifestyles and prevent diseases, which can eventually lead to the improvement of socioeconomic equality in health outcomes among the elderly.

As mentioned above, the contribution of this paper is to the research fields which are as follows: firstly, this paper focuses on the health inequality among rural residents rather than the whole China like existing paper. With the development of China, there are huge gaps among rural residents which could be different from others. This paper adds the evidence to the research of health inequality from this perspective. Secondly, in terms of method, in order to reduce the measurement bias, this paper transforms ordinal SAH status to cardinal HUI resort to the interval regression based on the threshold more suitable for China. Thirdly, using a panel database of 29,616 rural residents from 2010 to 2018, our results are more representative, more robustness, and more credible.

This study suffers from several limitations that warrant mention. Firstly, some important independent variables, which may significantly affect the health of the rural people, such as the regional air quality and the health insurance system, cannot be included in this study due to the unavailability of data. Secondly, the measurement of health in this paper concerns SAH, some measurements, such as objective health status and mental health status, are not included. We will conduct a more comprehensive measurement in the forthcoming research. Thirdly, although this paper confirms that there is a spatial correlation among different regions in China, we do not underly the mechanism. However, the aim of this paper explores the temporal and spatial changes of health inequality in rural China, we will inform the mechanism in the forthcoming research.

## Conclusions

Recently, health inequality has become one of the main obstacles to public health. This paper explores the temporal and spatial changes of health inequality in rural China. The main findings of this paper are as follows:

First, the health status of rural residents in China shows a fluctuant trend from 2010 to 2018. The CI is positive from 2010 to 2018, which means that there exists the health inequality in rural China, and the degree of health inequality is gradually narrowing. Specifically, through concentrated index decomposition, it shows that from 2010 to 2018, health resources are more concentrated in rural residents with higher income and education. However, the CI related to the household assets turns negative in 2018, which means in 2018, the health resources are more concentrated in the individual with fewer household assets. One of the probable reasons may be the promotion of China's poverty alleviation strategy and the new rural medical insurance system.

Second, we describe the spatial variations of health inequalities. The results show that health inequality from 2010 to 2018 varies among the provinces. It generally shows a downward trend, which eventually forms a regional feature of diminishing from eastern, central, and western. Specifically, in 2010, the health inequality of rural residents in China gradually expands from west to east. In 2012, the high-value area is transferred from the coastal areas to the inland areas. In 2014, the worst inequality happened in Hubei (鄂) and the best condition is in Zhejiang (浙), Jiangsu (苏) 331 and Beijing (京). In 2016, the high-value areas of health inequality and the low-value areas are 332 staggered, and the high-value areas are transferred to the Hubei (鄂) in the north China. The low333 value areas are mainly distributed in Zhejiang (浙), Jiangsu (苏), and Beijing (京). In 2018, the health 334 inequality get better in every province. And the best three provinces are Shanxi (陕), Heilongjiang (黑) 335 and Beijing (京). The worst condition occurs in Guizhou (黔).

Finally, by using Moran's Index, we find that there is a high global spatial positive correlation among the health inequalities in 2010–2014 and 2018 and a negative correlation in 2016. The spatial correlation of health inequality means that there is a kind of agglomeration of health inequality in geographical location.

Especially, this paper finds that behind the health inequality is the difference in individual social-economics status. The policy recommendations of this paper are first, the spatial perspective of health inequality plays an important role in improving health inequality. Secondly, narrowing the inequality in social-economic status is the key to improving health inequality. Among them, improving the distribution of educational resources of rural residents is an important part.

## Data Availability Statement

Publicly available datasets were analyzed in this study. This data can be found here: http://www.isss.pku.edu.cn/cfps/.

## Author Contributions

JJ and ZW conceived of the presented idea. WH and YL performed the computations, verified the analytical methods, and analyzed the study data. JJ and ZW encouraged WH to investigate and supervised the findings of this work. All authors discussed the results and contributed to the final manuscript.

## Funding

Social Science Fund of Liaoning, Grant/Award Number: L16BGL038; National Social Science Fund of China, Grant/Award Number: 21AZD044; Liaoning BaiQianWan Talents Program, Grant/Award Number: 2018-73; Scientific Research Funding Project of Liaoning Provincial Department of Education, Grant/Award Number: WSNQN202028 and WSNZK202003; Youth Project of Humanities and Social Sciences, Ministry of Education of China (Grant/Award No. 18YJC790109).

## Conflict of Interest

The authors declare that the research was conducted in the absence of any commercial or financial relationships that could be construed as a potential conflict of interest.

## Publisher's Note

All claims expressed in this article are solely those of the authors and do not necessarily represent those of their affiliated organizations, or those of the publisher, the editors and the reviewers. Any product that may be evaluated in this article, or claim that may be made by its manufacturer, is not guaranteed or endorsed by the publisher.

## References

[1] Anonymous. China's Challenges: Health and Wealth. Lancet. (2006) 367:623. 10.1016/S0140-6736(06)68234-2 16503440

[2] Xie E. Income-related inequalities of health and health care utilization. Front Econ China. (2011) 6:131–56. 10.1007/s11459-011-0125-5

[3] Wang H Cheng Z Smyth R. Health outcomes, health inequality and Mandarin proficiency in urban China. China Econ Rev. (2019) 56:22. 10.1016/j.chieco.2019.101305

[4] Cai J Coyte PC Zhao H. Decomposing the causes of socioeconomic-related health inequality among urban and rural populations in China: a new decomposition approach. Int J Equity Health. (2017) 16:9. 10.1186/s12939-017-0624-9 28720105 PMC5516311

[5] McCartney G Popham F McMaster R Cumbers A. Defining health and health inequalities. Public Health. (2019) 172:22–30. 10.1016/j.puhe.2019.03.023 31154234 PMC6558275

[6] WHO. Economics of Social Determinants of Health and Health Inequalities: A Resource Book (2013). Available online at: https://apps.who.int/iris/bitstream/handle/10665/84213/9789241548625_eng.pdf

[7] Fang H Rizzo JA. Does inequality in China affect health differently in high- vs. low-income households? Appl Econ. (2012) 44:1081–90. 10.1080/00036846.2010.534076

[8] Xue M Lim JY. Investigation on the health inequality in China. Int Area Stud Rev. (2016) 20:175–94. 10.21212/IASR.20.2.8

[9] Baeten S Van Ourti T van Doorslaer E. Rising inequalities in income and health in China: Who is left behind? J Health Econ. (2013) 32:1214–29. 10.1016/j.jhealeco.2013.10.002 24189450 PMC3880577

[10] Li H Zhu Y. Income, income inequality, and health: evidence from China. J Comp Econ. (2006) 34:668–93. 10.1016/j.jce.2006.08.00518325651

[11] Gkiouleka A Huijts T Beckfield J Bambra C. Understanding the micro and macro politics of health: Inequalities, intersectionality & institutions—a research agenda. Soc Sci Med. (2018) 200:92–8. 10.1016/j.socscimed.2018.01.025 29421476

[12] Zhang T Xu Y Ren J Sun L Liu C. Inequality in the distribution of health resources and health services in China: Hospitals vs. primary care institutions. Int J Equity Health. (2017) 16:9. 10.1186/s12939-017-0543-9 28253876 PMC5335774

[13] Bakkeli NZ. Income inequality and health in China: A panel data analysis. Soc Sci Med. (2016) 157:39–47. 10.1016/j.socscimed.2016.03.041 27060540

[14] Wang H Yu Y. Increasing health inequality in China: an empirical study with ordinal data. J Econ Inequal. (2016) 14:41–61. 10.1007/s10888-015-9315-1

[15] Pearce J Dorling D. Increasing geographical inequalities in health in New Zealand, 1980–2001. Int J Epidemiol. (2006) 35:597–603. 10.1093/ije/dyl013 16455757

[16] Wagstaff A. Inequality aversion, health inequalities and health achievement. J Health Econ. (2002) 21:627–41. 10.1016/S0167-6296(02)00006-1 12146594

[17] WAGSTAFF A VANDOORSLAER E. Measuring inequalities in health in the presence of multiple-category morbidity indicators. Health Econ. (1994) 3:281–91. 10.1002/hec.4730030409 7994327

[18] Fang P Dong S Xiao J Liu C Feng X Wang Y. Regional inequality in health and its determinants: evidence from China. Health Policy. (2010) 94:14–25. 10.1016/j.healthpol.2009.08.002 19735959

[19] Yang M Rosenberg MW Li J. Spatial variability of health inequalities of older people in china and related health factors. Int J Env Res Pub He. (2020) 20:17. 10.3390/ijerph17051739 32155968 PMC7084825

